# SUCCESSFUL APPROACHES TO ENHANCING OLDER ADULT REPRESENTATION IN MOTRPAC TRIAL

**DOI:** 10.1093/geroni/igae098.0406

**Published:** 2024-12-31

**Authors:** Sara Espinoza, Paul Coen, Jennifer Talton, Joseph Houmard, Kerrie Moreau, Barbara Nicklas

**Affiliations:** Cedars-Sinai Medical Center, Los Angeles, California, United States; AdventHealth, Orlando, Florida, United States; Wake Forest University, Winston-Salem, North Carolina, United States; East Carolina University, Greenville, North Carolina, United States; Colorado University, Aurora, Colorado, United States; Wake Forest University School of Medicine, Winston-Salem, North Carolina, United States

## Abstract

NIH mandates that clinical research include participants of all ages (or specify a reason for excluding based on age); yet, older adults (OAs) are often underrepresented even in studies without an age exclusion. Barriers to their inclusion are multi-factorial and include lack of recruitment strategies targeted to this demographic and eligibility criteria associated with age that reduce representation. Supported by multiple NIH Institutes, including NIA, the purpose of MoTrPAC is to characterize the molecular changes that underlie the benefits of exercise in humans. The goal is to randomize approximately 1980 sedentary, healthy participants (across 10 sites) to a 12-week control group or a supervised, moderate-intensity resistance or aerobic exercise intervention. The target is to balance enrollment across three groups (18-39, 40-59, and 60+ years). Recruitment is ongoing; as of 3/1/24, the number randomized is n=1043 with 20.6% OAs, compared to 40.5% younger and 38.9% middle-aged. Recruitment is monitored regularly, and early in recruitment a large age differential in eligibility yields was discovered, with only 4.5% of screened OAs being randomized, compared to 13.8% of middle-aged and 18.3% of younger individuals. Since the use of lipid-lowering medication was the single largest reason OAs did not qualify (36% of exclusions involved statin use in those >60 yrs), the protocol was altered to drop this exclusion to enhance OA representation. Also, successful recruitment marketing approaches are tracked closely and vary across age groups. This presentation will discuss the strategies which provide the most success for attracting OAs to inquire about the study.

